# The Role of MicroRNAs in Kidney Disease

**DOI:** 10.3390/ncrna1030192

**Published:** 2015-11-18

**Authors:** Sydwell Mukhadi, Rodney Hull, Zukile Mbita, Zodwa Dlamini

**Affiliations:** 1 Forensic Science Laboratory, 730 Pretorius street, Arcadia 0083, South Africa; E-Mail: mukhadist@gmail.com; 2 College of Agriculture and Environmental Sciences, University of South Africa, Private Bag X6, Florida 1709, Johannesburg 1709, South Africa; E-Mail: hullr@unisa.ac.za; 3 Department of Biochemistry, Microbiology and Biotechnology, University of Limpopo, Private Bag x1106, Sovenga 0727, South Africa; E-Mail: zukile.mbita@ul.ac.za; 4 Research, Innovation & Engagements Portfolio, Mangosuthu University of Technology, Durban 4031, South Africa

**Keywords:** miRNAs, kidney diseases, gene regulation, diagnostics, therapeutics

## Abstract

MicroRNAs (miRNAs) are short noncoding RNAs that regulate pathophysiological processes that suppress gene expression by binding to messenger RNAs. These biomolecules can be used to study gene regulation and protein expression, which will allow better understanding of many biological processes such as cell cycle progression and apoptosis that control the fate of cells. Several pathways have also been implicated to be involved in kidney diseases such as Transforming Growth Factor-β, Mitogen-Activated Protein Kinase signaling, and Wnt signaling pathways. The discovery of miRNAs has provided new insights into kidney pathologies and may provide new innovative and effective therapeutic strategies. Research has demonstrated the role of miRNAs in a variety of kidney diseases including renal cell carcinoma, diabetic nephropathy, nephritic syndrome, renal fibrosis, lupus nephritis and acute pyelonephritis. MiRNAs are implicated as playing a role in these diseases due to their role in apoptosis, cell proliferation, differentiation and development. As miRNAs have been detected in a stable condition in different biological fluids, they have the potential to be tools to study the pathogenesis of human diseases with a great potential to be used in disease prognosis and diagnosis. The purpose of this review is to examine the role of miRNA in kidney disease.

## 1. MicroRNAs: Discovery, Classification and Physiological Function

MicroRNA (miRNA or miRs) are small regulatory RNAs of approximately 19–25 nucleotides in length and are involved in post-transcriptional gene silencing in all eukaryotes [1,2,3]. They were discovered in 1993 in the nematode *Caenorhabditis elegans* [4] where they were shown to play crucial roles in gene regulatory networks. Their significance remained undervalued due to their unusual characteristics and unknown function at that time. After the discovery of the first miRNA, *lin-4*, in the nematode *C. elegans* [4], a second important miRNA, *let-7* was also identified in the same organism in 2000 [5]. The discovery of RNA interference (RNAi) revolutionized the understanding of gene regulation and led to the identification of several classes of small RNAs involved in gene regulation [6].

MiRNAs have been identified in many organisms. Approximately 17,000 miRNAs have been identified thus far with more than 1900 being found in humans [7]. MiRNA expression has been implicated as having fundamental roles in a variety of biological processes, such as differentiation, apoptosis and cell proliferation [4,8,9]. Most miRNAs are located in regions of the genome that are distant from locations of previously known genes. At least 30% of all human genes have been shown to be regulated by miRNAs and each miRNA may control hundreds of gene targets [10]. Target sites for miRNA binding are commonly found in the 3'-untranslated region (UTR) but can be found in the 5'-UTR or the coding region of the target mRNAs as well [11,12,13].

MiRNAs have the ability to silence a gene through partial binding to its complementary mRNA. The current view on the function of miRNAs is that they negatively regulate gene expression [14]. As negative and post-transcriptional regulators of gene expression, they bind partially to complementary sites of mRNAs at the 3'-UTR region and cause inhibition of translation or, in most cases, directing degradation of the bound mRNA, resulting in decreased mRNA levels [9]. Imperfect base pairing of miRNA inhibits the translation of their target mRNAs [6].

Increasing evidence has implicated miRNAs in a wide variety of human diseases such as liver, neurodevelopmental and cardiovascular diseases [15,16,17]. MiRNAs are also reported to play a role in disease pathogenesis through gene regulation of disease-related genes (Table 1).

**Table 1:** Different miRNAs involved in a variety of diseases other than renal diseases.

Diseases	MicroRNA	Gene Target	Pathway Affected	References
**Cancer**				
Chronic lymphocytic leukemia	miR-15a and miR-16	*BAZ2A*, *RNF41*, *RASSF5*, *MKK3* and *LRIG1*		[18]
Human hepatocellular carcinoma	miR-221	CDKN1B	Cell cycle	[19]
Breast cancer	miR-21	PDCD4	Apoptosis	[20]
Lung cancer	let-7 miRNA	CDK1	Proliferation	[21]
Pancreatic cancer	miR-34a	p53	Apoptosis	[22]
Neuroblastoma	miR-34a	BCL2, MYCN	Apoptosis, Proliferation	[23]
Human colon cancer	miR-145	IRS-1	Growth and Proliferation	[24]
Esophageal cancer	miR-21	Ran	Growth and Proliferation	[25]
**Vascular Disease**				
Myocardial infarction	miR-29	collagens, fibrillins, and elastin	Fibrosis proteins	[26]
Peripheral arterial disease	miR-221	Kip1 and Kip2	High glucose-induced endothelial dysfunction	[27]
Cardiac failure	miR-1	Bcl-2	Ischematic heart tissue	[28]
**Obesity**				
	miR-143	ERK5	Differentiation	[29]
**Amyotrophic Lateral Sclerosis (ALS)**				
	miR-23a	peroxisome proliferator-activated receptor γ coactivator-1α (PGC-1α)	Dysregulation in mitochondrial gene expression	[30]
	miR-29b		Muscle regeneration	[30]
	mir-455		Muscle wasting	[30]

## 2. MicroRNA Biogenesis

**Figure 1:**
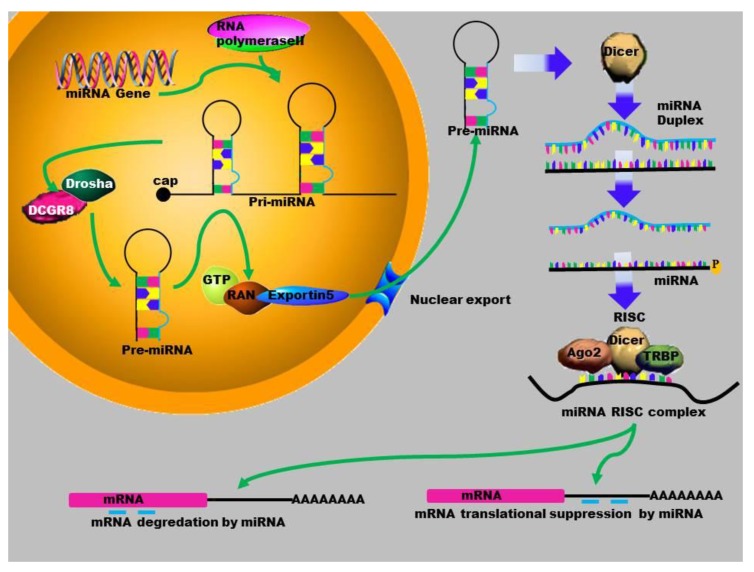
Schematic outline of the biogenesis of miRNA: MiRNA is transcribed by RNA polymerase II to form a primary microRNA molecule. These Pri-mRNA molecules are large RNA precursors and are comprised of a 5ʹ-cap and poly-A tail. This is then cleaved by the Drosha complex to generate pre-miRNA that is exported to the cytoplasm by Exportin-5, where it is processed by Dicer into a miRNA duplex. The guide strand (mature miRNA) is then incorporated into the miRISC where gene silencing can be accomplished via mRNA target cleavage (degradation), or through the prevention of translation.

Mature miRNAs are evolutionarily conserved single-stranded RNAs. Many miRNAs are encoded by genomic regions which are located within introns and intergenic regions of non-coding RNAs [31]. The biogenesis of miRNAs starts in the nucleus, where the miRNA gene is transcribed by RNA polymerase II or III, to produce a long primary miRNA (Figure 1). The MiRNA biogenesis pathway requires two RNase III enzymes, Drosha and Dicer. Drosha processes the primary miRNA transcript (pri-miRNA) into a ~60–100 nucleotides (nt) hairpin structure termed the precursor-miRNA (pre-miRNA). MiRNAs are transported out of the nucleus by Exportin-5 to the cytoplasm where they are further processed by another RNase III, Dicer, into mature, 22 bp nucleotides. One strand is degraded while the other one binds to the 3'-untranslated region of the target messenger RNA (mRNA) [32]. This complex is composed of several proteins that include the Argonaute proteins, which allow a stable conservation of the miRNA [2]. The interaction between the miRISC complex and the mRNA can also have a direct effect on protein translation. To date, several studies have shown that miRNAs also play a significant role in gene activation [33,34].

## 3. Gene Activation

Growing evidence is emerging that supports miRNAs as playing a significant role in regulating gene activation [35]. MiRNAs also regulate gene expression through target cleavage and/or translational inhibition. Multiple studies have shown that miRNA may also function to induce gene expression through different mechanisms under certain cellular conditions [13,33,36]. It was reported that miRNA-205 specifically activates the tumour suppressor genes IL24 and IL32 by targeting specific sites in their promoters [37]. This would suggest that the ability of miRNA to activate a gene would depend on the type of gene and the diseases with which they are associated.

MiRNA functions are not limited to actions in the cytoplasm, but can also perform tasks in the nucleus [38]. MiRNA can regulate transcription, by causing chromatin remodelling or DNA methylation of promoter sites. This process can alter the expression of gene targets and also cause gene activation [33,34]. The ability of miRNAs to concurrently down-regulate transcripts by post-transcriptional gene silencing mechanisms and potentially up-regulate target genes, suggests that they have a more complex nature and are of fundamental importance in regulating gene expression [33]. The ability of some miRNAs to induce gene expression through targeting the promoter sequence of target genes, is demonstrated by miR-373 which has a target site within the promoter sequence of E-cadherin and cold shock domain containing protein (CSDC2) and induced expression of these proteins. Moreover, enrichment of RNA polymerase II was detected at both promoter sites after miR-373 transfection [33].

## 4. MicroRNAs in Human Diseases

Approximately 20,000 annotated protein-coding genes have been reported to exist in the human genome, with over 2400 candidate miRNA genes [39]. However, this constitutes only 2% of the genome and more than 70% of the human genome is transcribed into non-protein coding transcripts, which was previously considered as genomic junk or noise [40]. It has been demonstrated by many groups how miRNAs regulate disease pathways (summarised in Table 1). MiRNAs have been reported to be involved in in the pathogenesis of many human diseases [3,41] through their involvement in the regulation of biological processes implicated in disease progression including apoptosis, development, proliferation and differentiation [42,43]. Many studies have been conducted on miRNAs and their correlations with various diseases such as cancer of different organs [44,45]. These also suggest that miRNAs play a significant role in the functioning of the kidneys. The abnormal expression of miRNAs in different diseases may be caused by mutation in miRNA encoding genes or aberrant miRNA biogenesis.

It has been demonstrated that almost 50% of human miRNA genes are located in cancer associated regions [46]. For example, miR-16a expression (at 13q14) has been reported to be low or absent in the majority of B-cell chronic lymphocytic leukaemia (B-CLL), [46]. A role played by miRNAs in several types of cancer has been well documented [47]. This includes Chronic Lymphotic Leukaemia (CLL) where expression of miRNAs, miR-15a and miR-16 was found to be down-regulated [48].

Reduced levels of miR-25 in the kidneys from both diabetic rats and high glucose-treated mesangial cells indicated that miR-25 may regulate NOX4 expression and function in diabetic nephropathy [49]. In a rat model of myocardial infarction and human coronary heart disease, miR-1 was significantly down-regulated in ischemic heart tissue [50].

Recently, it has been demonstrated that miRNAs play a critical role in renal development, maintenance of renal function and progression of kidney diseases [44,51,52]. However, it is now known that miRNAs can regulate entire networks of genes and are considered as master regulators of the human genome [53,54]. MiRNAs regulate patho-physiological pathways by post-transcriptionally inhibiting the expression of a plethora of target genes [55]. Many studies have been made on the role of miRNAs in human diseases, especially in cancer [56,57,58]. Since a single miRNA can have many target genes, multiple pathways can therefore be affected in diseases [9,59]. Several pathways have been implicated to contribute to the pathogenesis of renal diseases such as TGF-β, MAPK signalling, and Wnt signalling pathways [1,60,61]. In this review we will focus on the role of miRNAs expressed in different kinds of kidney diseases.

## 5. MicroRNA in Renal Diseases

Kidney and urinary tract diseases are common disorders that cause approximately 830,000 deaths annually. This ranks kidney diseases as the 12th most common cause of death accounting for 1.4% of all deaths [61]. Numerous studies have implicated the involvement of miRNA with various kidney diseases such as, chronic kidney diseases (CKD). Chronic kidney diseases occurs when disorders such as fibrosis, diabetes, hypertension, inflammation or toxic substances eventually lead to kidney failure. MiRNAs have emerged as important post-transcriptional regulators of gene expression in renal diseases, where they play a role in the regulation of fundamental cellular activities such as development, differentiation, proliferation, apoptosis, immune regulation and organogenesis [62]. A significant amount of research associated with miRNA expression within the kidney is focused on regulation and functioning in various renal diseases [62]. Several studies have demonstrated a critical role for miRNAs in renal development, physiology, and pathophysiology [62,63,64]. Several miRNAs are specific to certain tissues or stages of development, indicating that they could play important roles in many biological processes. MiR-377 has been shown to be up-regulated in both mouse and human mesengial cells [65]. Identification of miRNA regulation and function in renal pathophysiology may lead to the exploitation of miRNAs as new therapeutic targets in various kidney diseases.

### 5.1. MicroRNAs in Diabetic Nephropathy (DN)

Diabetic nephropathy (DN) is a progressive kidney disease that can lead to end stage renal diseases (ESRD) due to complications, arising from diabetes. It is characterised by the accumulation of extracellular matrix (ECM) proteins, glomerular basement membrane thickening, mesangial expansion and hypertrophy [66]. Since the discovery of miRNA, various studies have demonstrated the potential role of miRNA in diabetic nephropathy. An early study described the role of miRNA in diabetic nephropathy, identified through the control of Transforming Growth Factor-β (TGF-β) expression by miRNA as a key factor in diabetic nephropathy [66]. TGF-β has been implicated as a key mediator of mesangial matrix deposition and recent research has demonstrated that TGF-β-mediated miRNA regulation is involved in diabetic nephropathy [67].

### 5.2. MicroRNAs in Renal Fibrosis

Fibrosis is the leading cause of organ dysfunction in diseases, either as the outcome of an uncontrolled reaction to chronic tissue injury or as the primary disease itself in predisposed individuals [68]. Renal fibrosis can be defined as the excessive accumulation of extracellular matrix that leads to end stage renal failure. As in diabetic nephropathy, TGF-β is regarded as a critical regulator of several miRNAs in renal fibrosis and is a key pathological mediator of fibrotic diseases. A recent study suggested that TGF-β promotes renal fibrosis by inducing renal miR-433 expression [69]. The TGF-β/Smad pathway is one mechanism by which miR-21 expression is increased in fibrotic tissues. It has recently been reported that there is differential transcription of miR-205 and miR-192 in IgA nephropathy, and these changes correlate with disease severity and progression [70].

### 5.3. MicroRNAs in Lupus Nephritis (LN)

Lupus Nephritis (LN) is a common and severe outcome-defining complication in Systemic Lupus Erythematosus (SLE) affecting up to 60% of patients at some point of the disease. MiRNA expression profiles of renal tissue have gained much attention since Dai *et al.* [71] provided a broad analysis of differentially expressed miRNAs in lupus nephritis kidney biopsy samples from 11 patients with three controls *in vivo*. Furthermore, miR-423 and miR-663 were demonstrated to be down-regulated in lupus nephritis [72]. This suggests that miRNA expression patterns are cell and organ specific. The levels of many miRNAs, such as miR-200a, miR-200c, miR-141, miR-429 and miR-192, are lower in patients with active lupus nephritis than those in healthy controls [73]. Another study reported the differential expression of miR-638, miR-663, miR198, miR-155, and miR-146a from dissected glomeruli, tubules and interstitial tissues from kidney biopsy [73]. Glomerular tissue from lupus nephritis patients showed an increased expression of miR-146a, lupus nephritis tubule-interstitial tissue did not [74]. Finally, it has been shown that different sets of miRNAs are differentially expressed in lupus nephritis patients from different racial groups. This emphasizes the importance of miRNAs as biomarkers for the diagnosis or prognosis of lupus nephritis [74]. Further support for this is the fact that a combination of miR-192 and miR-27b from urinary exosomes could differentiate lupus patients with or without nephritis [75] while the levels of miR-221 and miR-222 in urinary sediment are inversely correlated with serum anti-dsDNA level in patients with active lupus nephritis [76].

### 5.4. MicroRNA in Polycystic Kidney Diseases (PKD)

Polycystic kidney disease is a leading cause of end stage renal failure and is the result of the progressive growth of renal cysts. The mechanisms that lead to cyst formation in the kidney are due to the deregulated expression or mutation of the causative genes polycystic kidney disease 1 (PKD1), polycystic kidney disease 2 (PKD2) and polycystic kidney and hepatic disease 1 (PKHD1) [62]. The mutations can be either autosomal dominant or autosomal recessive. PKD is characterised by uncontrolled protein translation during cell division. About 80% of adults with autosomal dominant polycystic kidney disease (ADKP) are diagnosed with hypertension before the loss of kidney function [77]. The involvement of miRNAs in PKD was first demonstrated in a rat model, as rats or mice have been used as common model systems for polycystic kidney disease. These models suggest that miRNAs could play a significant role for therapy and diagnosis of the disease [78]. Differential expression of miRNAs were observed in the kidneys of a PKD rat model, where the de-regulated miRNAs targeted many signalling pathways, (mTOR signalling, mitogen-activated protein kinase signalling, Wnt signalling and the TGF-β pathway) involved in PKD with the abnormal expression of these genes leading to disrupted cell division and proliferation [79].

Pandey *et al.* [80] discovered that 30 miRs in the kidney were differently expressed in a rat model, with only miR-21 and miR-217 not being previously identified in the kidney [80]. Another study shows that miR-17 directly targets PKD2 and could also have a role in cytogenesis [81]. Additionally, it was shown that miR-15 could repress the expression of cell division cycle 25A (Cdc25A), resulting in inhibition of the cell cycle and proliferation. Reduction in miR-15 transcription promotes the increased expression of Cdc25A, thus promoting cyst growth in disease conditions [82]. The expression of the PKHD 1 gene is inhibited by miR-365-1, resulting in suppressed cell adhesion in PKD.

### 5.5. MicroRNA in Renal Cell Carcinoma (RCC)

Renal cell carcinomas (RCC) are a family of carcinomas that originate from the epithelium of the renal tubules and accounts for approximately 90% of all renal malignancies [83]. Renal cell carcinoma is the most invasive and common neoplasm of adult kidney, representing 2%–3% of adult malignancies. Clear cell renal cell carcinoma (ccRCC) is the early stage of renal cancers which is clinically asymptomatic resulting in difficulties establishing an accurate diagnosis. Aberrant expression of miRNAs has been reported to be associated with different types of cancers and to play a vital role in the progression of renal cancer. 

**Figure 2:**
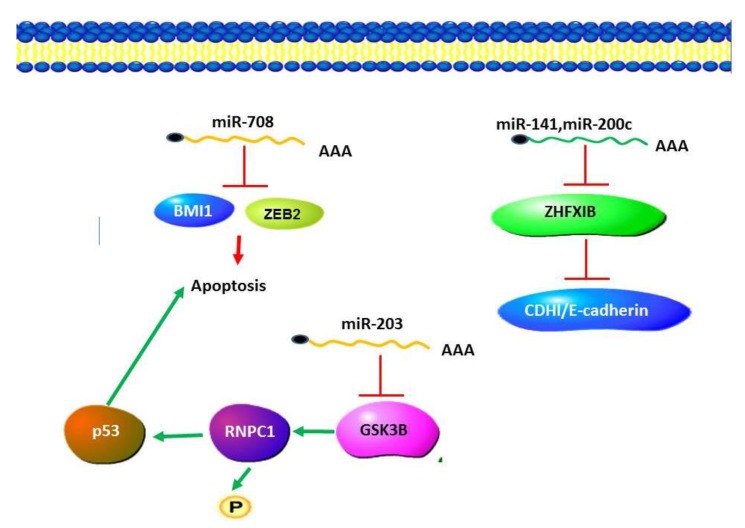
MicroRNA in Renal Cell Carcinoma (RCC). miR203 plays an anti-apoptotic role by negatively regulating the expression of GSK-3β, resulting in a decrease in the levels of p53. Apoptosis is induced by the activity of miR708 which functions to target BMI1 and ZEB2. Cancer progression is promoted by a decrease in the levels of miR-141 and miR-200c, which results in increased ZFHX 1B levels and a decrease in cadherin levels.

A number of miRNA expression studies have been carried out in renal cell carcinoma including oesophageal squamous cell carcinoma (ESCC), comparing miRNA expression profiles between RCC and normal kidney tissues [84,85,86]. These studies indicated that miR-424 is associated with RCC [84]. A number of studies have reported down-regulation of miR-203 in various types of cancers [87,88,89]. MiR-203 has been reported to function by targeting GSK-3β to activate pathways in RCC [90]. GSK-3β was found to promote p53 mRNA translation via phosphorylating RNPC1. It may be that miR-203 is one of the factors either driving or resulting from this progression by an epigenetic mechanism or by other biological processes. MiR-708 has been reported to target BMI1 and ZEB2 and induce apoptosis in cellular pathways [87]. This miRNA could potentially regulate apoptosis and cell proliferation (Figure 2).

### 5.6. MicroRNAs in Wilm’s Tumor

Wilm’s tumor (WT, also known as nephroblastoma) is a renal cancer that arises predominantly in children and rarely in adults, and is thought to develop from the embryonic kidney [88]. Multiple studies have examined miRNA expression in Wilms’ nephroblastoma [89,90,91]. Several mutations have been identified in Dicer1 (the key miRNA-processing enzyme) in patients with Wilm’s tumor. Oncomir is the term given to miRNAs that are associated with cancer, with the oncomir-1 gene giving rise to multiple oncomirs. The expression of this gene was found to be significantly up-regulated in patients with Wilm’s tumor by the transcription factor E2F3 [89].

The oncogenetic transcription factor STAT3 is able to up-regulate miR-370 expression which down-regulates the level of FAM123B, which regulates the Wnt signalling pathway. MiR-562 is implicated in targeting eyes absent homolog1 (EYA1) within the 2q37.1 region [92]. EYA1 has been shown to be down-regulated in Wilm’s tumor. Studies have indicated that the expression level of miR483-3p was higher in Wilm’s tumour samples compared to the normal controls [93]. Expression of miRNA regulator of transcription factor (Six1), miR-185, is down-regulated in paediatric malignancies, including Wilm’s tumor [94]. Kort *et al.* [95] reported on the role of the miR-17-92 miRNA cluster in Wilms’ tumors. This cluster plays a prominent role in promoting tumor cell proliferation [90]. The miR-17-92 cluster is a cell cycle regulated locus and ectopic expression of a single miRNA, miR-17-5p, is sufficient to drive a proliferative signal [96]. MiR-193a silences the Wilms’ tumor (WT1) gene, which encodes a transcriptional factor and acts as a master regulator for podocyte homeostasis [97].

### 5.7. MicroRNAs in IgA Nephropathy

IgA nephropathy (IgAN) is the most common inflammatory diseases of renal corpuscles and is characterized by the presence of immunoglobulin IgA deposits in the kidney. Many studies have demonstrated that miRNAs play a significant role in the pathogenesis of inflammatory and immune disease including IgAN, rheumatoid arthritis, multiple sclerosis and systematic lupus erythematous [62,102,103,104]. Sixty-five miRNAs were differentially expressed in IgA nephropathy and roughly half were down-regulated [71]. Previous studies showed that levels of miR-146a and miR-155 in urine correlated with the levels of various inflammatory markers in IgA nephropathy [73,101]. Studies of the intra-renal expression of miRNAs in IgAN patients revealed that miR-200c was down-regulated while miR-141, miR-192 and miR-205 were up-regulated. Mature miR-196a expression has been shown to be down-regulated in IgAN patients [70].

It has also been speculated that the abnormal expression of miR-148b in peripheral blood mononuclear cells could account for the aberrant glycosylation of IgA1 in IgA nephropathy. A comprehensive microarray screening of miRNA in IgAN and revealed a pathophysiological mechanism whereby the miRNA 418b regulates the levels of mRNA encoding the C1β3GALT1 single nucleotide polymorphism (SNP) in IgAN patients [101]. The functional consequence of this is still controversial, as it is unknown whether IgA1 is under galactolysed as a consequence or of its reduced expression [102,103]. Patients with IgA nephropathy exhibited lower C1GALT1 expression, which negatively correlated with miR-148b expression. MiR-148b binds to the 3ʹ-untranslated region of C1B3GALT mRNA and breaks it down. The level of miRNA-148b expression is significantly higher in IgAN patients. C1β3GALT down-regulated by the Th2 cytokine, IL-4 and miR-418b may be induced by the Th2 cytokine. More recently, another study has reported differential transcription of miR-205 and miR-192 in IgA nephropathy correlates with disease severity and progression [70]. 

### 5.8. MicroRNAs in Renal Ischemia/Reperfusion

Ischemia-reperfusion injury (IRI) is a major causal factor of acute kidney injury and is associated with delayed graft function, chronic allograft injury and often results in death. Acute kidney injury (formerly known as acute renal failure) is a syndrome characterised by the rapid loss of the kidney’s excretory function [104]. Dicer knockout mice showed normal development, histology, and function of the kidney. These conditional KO mice were also found to be resistant to renal IRI, showing significantly better renal function, less tissue damage, lower tubular apoptosis rate, and higher survival rates [105].

Microarray analyses in wild type animals undergoing the same IRI procedure revealed changes in miRNA expression levels in the proximal tubule. One-hundred and seventy-three miRNAs were detected in the renal cortex, and miRNA-132, -362, -379, -668, and -687 showed continuous changes during 12–48 h of reperfusion [105]. Another study demonstrated similar changes in miRNA expression during renal IRI in laboratory mice [106].

p53, a pivotal protein in the apoptotic pathway, has been identified as a mediator of transcriptional responses of IR injury. The plasma of patients with acute kidney injury showed miR-16 and miR-320 down-regulated in comparison with healthy control individuals, while miR-210 showed significant up-regulation [107]. It has been demonstrated that the consistent expression level of miR-494 and Neutrophil Gelatinase-Associated Lipocalin (NGAL) can be used as an indicator of acute kidney injury (AKI) [108]. The first evidence of the involvement of miRNAs in AKI came from the observation that in proximal tubule-specific Dicer knockout mice, miRNAs were depleted [105], and changes in miR-21 regulation have been reported by various groups following both acute as well as chronic models of kidney injury [109,110]. 

### 5.9. MicroRNA in Allograft Acute Rejection

Acute rejection is a life-threatening complication after renal transplantation. In a study by Anglicheau, [111], 17 miRNAs were found differentially expressed in acute rejection biopsies. Twelve miRNAs were found to be down regulated in acute rejection after renal transplantation as compared with the controls, whereas eight miRNAs were up-regulated [112]. In another study 20 miRNAs were identified to be differently expressed in three patients with acute kidney allograft rejection [113], while a third study demonstrated that the up-regulation of the following miRNAs: miR-182, miR-155, miR-125a, miR-146b, was associated with antibody mediated rejection [114]. Finally, patients with acute rejections that were diagnosed by a kidney biopsy, showed reduced urinary levels of miR-210 [115]. 

Twenty miRNAs are differentially expressed in acute rejection allografts, of which 12 were de-regulated and eight up-regulated in AR, when compared with normal allograft biopsies [116]. Currently, the functional significance of the changes has not been studied; however, differentially expressed miRNAs can be targeted to prevent acute rejections from renal transplants. Among these miRNAs, there are three up-regulated miRNAs (miR-142-5p, miR-155 and miR-223) and three down-regulated miRNAs (miR-10b, miR-30a-3p and let-7c) that can be regarded or used as diagnostic hallmarks of AR [111]. MiR-210 levels are associated with acute renal allograft rejection, suggesting that it may serve as a novel biomarker of AR [117]. Accurate diagnoses and effective treatments of AR will enormously reduce the mortality rates of renal transplant patients. 

### 5.10. MicroRNA in Nephrotic Syndrome

Nephrotic syndrome is associated with an increase in permeability across the glomerular filtration barrier due to processes that affect the dynamics and permselectivity of glomerular filtration. It is characterised by distinct clinical abnormalities, such as proteinuria in the nephrotic range, hypoalbumenia, edema, and hyperlipidemia. The urinary level of miR-638 is reduced in adult patients with nephrotic syndrome irrespective of the underlying pathology [108], while the serum concentrations of miR-30a-p were increased in nephrotic syndrome [116]. The concentrations of multiple miRNAs (miR-30-5p, miR152-3p, miR150, miR-191 and miR19b) are altered in the serum and the urine of patients with nephrotic syndrome. Many of these miRNAs showed increased expression in diseased patients. This increase in expression was also found to decrease as a result of successful treatment [116].

### 5.11. MicroRNA in Human Immunodefiency Virus Associated Nephropathy (HIVAN)

Renal diseases have been increasingly recognised as the most important common complication of Human Immunodeficiency Virus (HIV) infection worldwide. One of the most common kidney diseases in patients with HIV/AIDS is human immunodeficiency virus-associated nephropathy (HIVAN), which is an end-stage renal disease (ESRD) [118]. HIVAN was initially described in patients with Acquired Immunodeficiency Syndrome (AIDS) [119] and was previously known as AIDS-associated nephropathy. However, the name was changed to HIVAN when renal histological features similar to those observed in patients with full-blown AIDS were observed in asymptomatic individuals. HIVAN can be defined as an aggressive form of focal segmental glomerulosclerosis (FSGS) characterized by collapse of the glomerular tuft and associated tubule interstitial lesions, and develops late in the course of HIV-1 infection following the development of AIDS [120,121]. 

The levels of certain miRNAs have been observed to increase or decrease during HIV infection implying that miRNAs play a significant role in HIV associated diseases [122]. Additionally, the deregulation of miRNAs during HIV infection has been observed to play a crucial role in disease development and progression [123]. Different studies have shown that deregulation of miRNAs due to HIV-1 infection plays a crucial role in disease development [122,124], and provided evidence that miRNAs may be early signs of host cellular dysfunction induced by HIV-1. In one such pathology, HIV encephalitis (HIVE), changes in miRNA regulation led to an increase in the levels of apoptosis contributing to the disease pathology [125].

### 5.12. MicroRNA in Hypertensive Nephrosclerosis

Chronic hypertension can result in kidney damage leading to hypertensive kidney disease or hypertensive nephrosclerosis. Hypertensive nephrosclerosis is a disorder that is usually associated with chronic hypertension and is characterised histologically by vascular, glomerular, and tubulointerstitial involvement [126]. However, the exact molecular mechanism that leads to hypertensive nephrosclerosis remains unknown. The examination of 34 patients with hypertensive neprhosclerosis, and controls from normal renal tissue, led to the identification of a number of miRNAs whose expression was increased. These included miR-200a, miR-200b, miR-14, miR-429, miR-205, and miR-192 [70].

While these differences in the miRNA expression are interesting, further work needs to be performed on the identification of miRNAs and target genes in hypertensive nephropathy. In another animal model, nephrectomy induces hypertension, associated with TGF-b pathway activation. The expression of 60 genes and 24 miRNAs was found to be altered in human hypertensive kidneys [127]. These included genes such as nuclear receptor sub-family 4 group A member-1,-2 and -3 (NR4A1, NR4A2, NR4A3), period circulation protein homolog 1 (PER1), and salt-inducible kinase 1(SIK1) as well as Renin. This implies that miRNAs play a crucial role in the expression of these genes in hypertensive nephrosclerosis [127].

### 5.13. MicroRNA in Acute Pyelonephritis (APN)

Acute pyelonephritis is the most common bacterial and life-threatening form of urinary tract infection (UTI) that causes irreversible kidney damage, followed by renal failure. An earlier study by Rollino *et al.* [128] has revealed that it is often difficult to characterise the severity of acute nephritis with clinical parameters. Allograft biopsy and urine culture are the best tools currently available to diagnose acute nephritis in the renal allograft, several of these biopsies also had overlapping features of acute nephritis and acute rejection. A panel of 25 miRNAs whose expression significantly differed between rejection and acute pyelonephritis were identified [129]. Five of these miRNAs (miR-145, miR-99b, let-7b-5p, miR23b, and miR-30a) were found, using qPCR, to be down-regulated in acute rejection as compared with normal kidney and acute pyelonephritis. MiR-23b was found to suppress several pro-inflammatory signalling pathways including IL-17, tumor necrosis factor α, IL-1-induced NF-κB, TGF-β-activated kinases and several others in human lupus and rheumatoid arthritis as well as in a mouse model [130]. This suggests that pro-inflammatory signalling pathways are associated with APN.

Interestingly, miR-145 and miR-99b have been implicated in neutrophil differentiation and are involved in the temporal expression of genes in the different stages of myeloid maturation. In a recent study expression of miR-145 and miR-99b was mildly increased in acute pyelonephritis biopsies compared with baselines, which may be a manifestation of neutrophil-predominant inflammation in acute pyelonephritis. Recent studies also show that biopsy of APN and AR are overlapping and difficult to distinguish [129].

### 5.14. Drug Associated Nephrotoxicity

Many of the classes of drugs used to treat ailments or for diagnostic purposes can be toxic to the kidney. It is suspected that 17%–26% of Acute Kidney Injuries that occurs during hospitalisation is due to drug related toxicity [130]. The nature of the functions performed by the kidney exposes the organ to a greater risk of damage caused by drugs. Firstly the kidneys are exposed to high levels of the drug and its metabolites due to the high level of blood flow the kidneys receive. Secondly many drug or metabolite particles that are the correct size and charge can enter the renal epithelial tubular cells. These cells are at great risk due to the high metabolic activity. Thirdly, as water is reabsorbed the effective concentration of these drugs increases, possibly to toxic levels. Fourthly, the oxidation of drugs by cytochrome p450 generates reactive oxygen species. Finally the pH conditions of the kidney may lead certain compounds to form insoluble crystals that can cause physical damage [131].

The toxicity of many drugs may be an inherent characteristic of the compound. Chemotherapy drugs such as cisplatin are a good example of drugs whose use can be limited due to nephrotoxicity. Cisplatin functions by causing DNA crosslinking, leading to apoptosis. The transcription of mir-155 increases in the kidneys following toxic injury and it is used as a biomarker for toxic injury. Mutant miR-155(−/−) mice treated with cisplatin had higher levels of kidney injury than normal control mice [132]. This response involved the activation of signalling pathways relating to apoptosis and oxidative stress; these pathways are regulated by c-Fos, which is directly regulated by miR-155 [132]. Other miRNAs that are affected by cisplatin include miR-122, which was down-regulated following cisplatin treatment, and miR-34a which was up-regulated following cisplatin treatment. Mir-122 inhibits Foxo3 translation, which normally activates p53. The down-regulation of mir-122 by cisplatin leads to an increase in Foxo3 activity, resulting in increased p53 levels and an increase in apoptosis [133]. Cyclosporine is another drug that can lead to nephrotoxicity by inducing tubular epithelial cell epithelial-mesenchymal transition (EMT). Treatment of mice with cyclosporine led to increased transcription of miR-494 and a decrease in PTEN levels *in vitro*. [134].

## 6. Transforming Growth Factor (TGF-β)

The cytokine Transforming growth factor (TGF-β) plays an important role in chronic kidney disease, and TGF-β has been reported to play a prominent role in glomerular cell proliferation and glomerular extracellular matrix expansion both of which contribute to renal failure [135]. miRNA and TGF-β co-ordinately regulate mitochondrial dysfunction, oxidative stress, and energy metabolism in oxidative stress-associated renal injury [66]. TGF-β1 acts by stimulating Smad3 to positively regulate miR-21 and miR-192, but negatively regulate the miR-29 or miR-200 families, to mediate renal fibrosis [64]. Apoptotic signalling by TGF-β occurs via members of the SMAD protein family of transcription factors, with different SMADs performing different functions. SMADS function as a trimer consisting of two receptor regulated SMADS (SMAD1, SMAD2, SMAD3, SMAD5 and SMAD8/9) and one co-SMAD (SMAD4). The system is regulated through the activity of inhibitory Smads (SMAD6 and SMAD7). TGF-β is recognised as a key mediator in the pathogenesis of renal fibrosis both in experimental models and in human kidney diseases [62].

**Figure 3:**
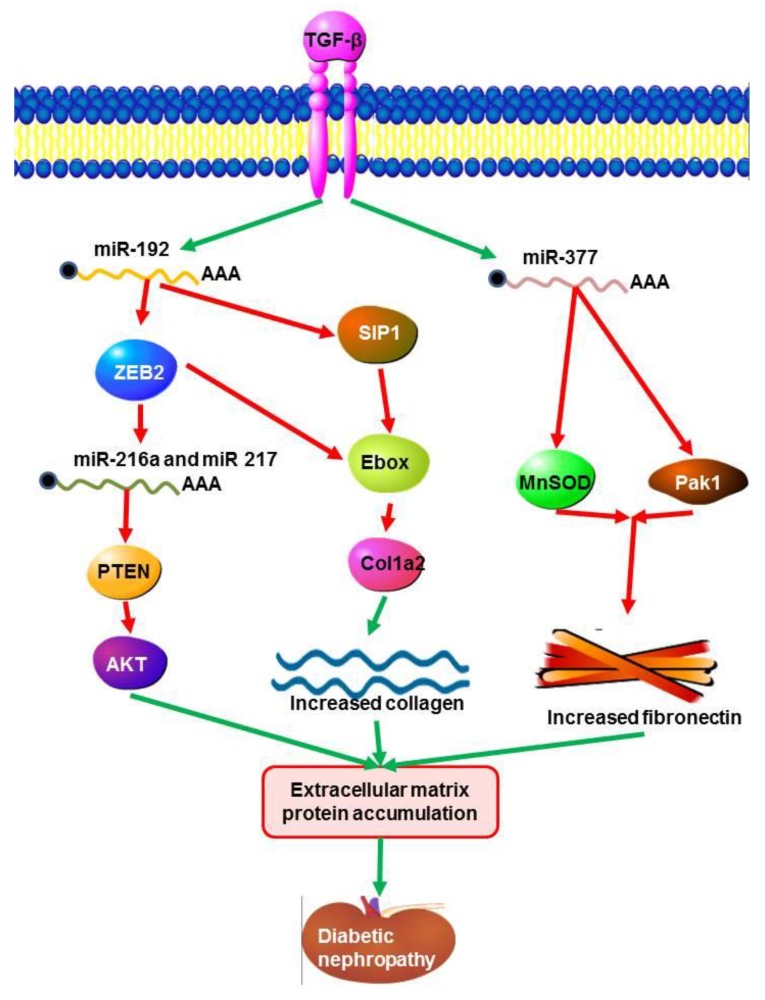
Signalling pathways initiated by miRNAs and TGF-β in diabetic nephropathy. The TGF-β signalling pathway is responsible for the induction of ECM matrix protein expression. This process is positively up-regulated through the activity of miR-192 which acts to negatively regulate E-box repressors (ZEB1 and ZEB2) and Smad-interacting protein 1 (SIP 1),which are responsible for down regulating the expression of induced extracellular matrix proteins during diabetic nephropathy. TGF-β regulates the expression of miR-216a and miR-217 through the down-regulation of phosphatase and tensin (PTEN) by AKT kinase. Fibronectin expression is increased through the down-regulation of p21-activated kinase (PAK1) and superoxide dismutase (MnSOD) by miR377a.

It has been recently reported that miR-192 regulates E-box repressors; (ZEB1 and ZEB2) that are responsible for controlling the expression of TGF-B induced extracellular matrix proteins during diabetic nephropathy [136]. TGF-β increases the expression of ECM proteins, such as collagens by reducing the E-box repressor and Smad-interacting protein 1 (SIP 1). Evidence shows that ECM genes are regulated by TGF-β through Smads in mesangial cells [142]. *MiR-192* and TGF-β increases the expression of miR-200 family members which can target Zeb1/2. Expression of miR-216a and miR-217 in mesangial cells in diabetic nephropathy were regulated by TGF-β1 through down-regulation of phosphatase and tensin (PTEN) by AKT kinase, a key modulator in diabetic nephropathy [138]. Mesangial cells stimulated with TGFB or by a high concentration of glucose, displayed up-regulation of miR-377 [65]. This increased transcription of miR-377 induced fibronectin (ECM protein) expression through suppression of p21-activated kinase (PAK1) and superoxide dismutase (MnSOD), which enhances fibronectin production (Figure 3) [65].

**Figure 4:**
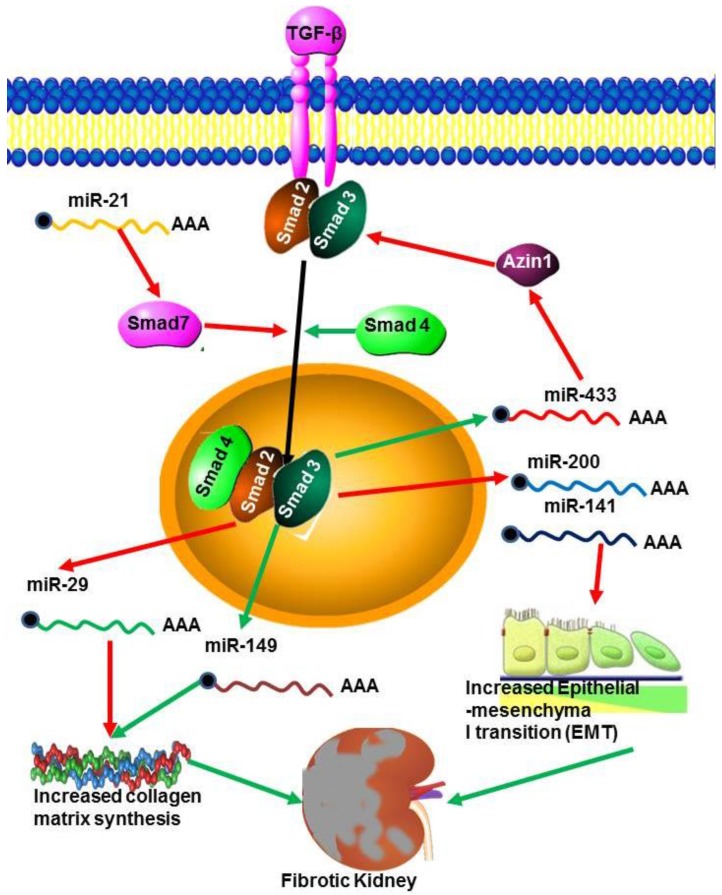
miRNAs in renal fibrosis. The TGF-β pathway is activated during renal fibrosis by the decrease in transcription of the Smad inhibitor Azin by miR-433. At the same time miR21 decreases the transcription of the inhibitory Smad7 leading to increased TGF-β signalling. However, miR29 inhibits collagen synthesis but the action of this miRNA is blocked by miR-149.

TGF-β is recognised as a key mediator in the pathogenesis of renal fibrosis both in experimental models and in human kidney diseases [62]. During renal fibrosis TGF-β signalling is enhanced through the expression of miR-433, which suppresses Azin-1, an antizyme inhibitor *in vitro* and *in vivo* (Figure 4). This suggests that miR-433 may be a critical fibrosis related miRNA in TGF-β/Smad3 driven renal fibrosis [69]. The miRNAs miR-200a and miR-141 are also, necessary for the development and progression of TGF-β1 dependent EMT and fibrosis *in vitro* and *in vivo* [62], and miR-192 up-regulation mediates activation of TGF-β/Smad signalling in the fibrotic kidney both *in vivo* and *in vitro* [139]. The TGF-β/Smad pathway is one mechanism by which miR-21 expression is increased in fibrotic tissues. For example, miR-21 expression was up-regulated in response to treatment with TGF-β1 or TNF-α in human renal tubular epithelial cells (TEC) *in vitro* [140]. These effects are mediated through attenuation of TGF- β extracellular matrix production, inflammation and epithelial metabolic pathways. miR-192 and TGF-β also increased the expression of miR-200 family members which can target Zeb1/2 [66]. Similarly, inhibition of miR-192 reduces TGF-β1-induced collagen accumulation in TEC, while overexpression of miR-192 enhances TGF-β1-induced collagen accumulation in TEC [7]. This suggests that miRNA regulates different genes in response to TGF-β signalling and may also be involved in regulating TGF-β expression.

## 7. Important miRNAs

### 7.1. miR-29a

Members of the miRNA 29 family are generally down-regulated in most types of cancers, although there are exceptions. Generally they regulate the expression of proteins such as collagens, transcription factors and methyltransferases [141]. A recent study demonstrated that increasing miR-29a levels protects cells against diabetic podocytopathy by suppressing HDAC4 signalling, nephrin ubiquitination, and urinary nephrin excretion associated with diabetes, as well as restoring nephrin acetylation [142]. TGF-β also down-regulates the expression of miR-29 [7,143], while miR-29 could inhibit TGF-/Smad3 mediated renal fibrosis only *in vitro* [144]. This miRNA also plays a role in kidney fibrosis, where it down-regulates fibrosis by targeting the processes of collagen matrix synthesis rather than by inhibiting myofibroblast accumulation [145]. The increased expression of miR-29b was implicated as playing a protective role in the renal medulla in non-salt sensitive rats. Several collagen, genes, such as matrix metalloproteinase 2 (Mmp2) and intergrin beta-1 (Itgb1) were found to be associated with up-regulation by miR-29b [123], with the inhibition of several of these collagen genes implicating that an increase in mir-29b levels may protect the rat from hypertensive nephropathy.

### 7.2. MiR-21

MiR-21 is not specific to a certain diseases and is believed to play a significant role in the progression of many malignancies, such as cancer and kidney diseases. Consequently, it is not surprising that miR-21 is involved in various biological processes, including cell differentiation, proliferation, and apoptosis. MiR-21 has been reported to be overexpressed in kidney diseases. However, of the thirty miRs identified as differently expressed in a rat model of PKD, only miR-21 and miR-217 had not been previously identified in the kidney [99]. MiR-21 has been implicated in playing a significant protective role in glomerular hypertrophy and early diabetic nephropathy. Previous studies showed that miR-21 prevented hypertrophy by targeting the phosphatase and tensin homolog/phosphatidylinositol-4,5-bisphosphate 3-kinase/Protein kinase B (PTEN/PI3K/Akt) pathway *in vivo* and *in vitro*, and over-expression of PTEN may act as an originator or modulator of diabetic nephropathy [146]. Over-expression of miR-21 inhibited proliferation of mesangial cells in high glucose conditions. Additionally, it was reported that Smad7 and AKT1 substrate 1(PRAS40), a negative regulator of Tor complex 1 (TORC1), are potential targets of miR-21 [147].

It has been reported that suppression of miR-21 reduces renal fibrosis in rodent kidney disease models [148]. MiR-21 expression increases in the kidneys of mice subjected to unilateral ureteral obstruction (UUO) or ischemic reperfusion injury (IRI), the two well established animal models of kidney fibrosis, and inhibition of miR-21 attenuates kidney fibrosis in mouse models. The role of miR-21 in renal fibrosis is further evident during the interaction of this miRNA with TGF-β/Smad signalling. A recent study suggested that TGF-β promotes renal fibrosis by inducing renal miR-433 expression [69]. Additionally, it was reported that two miRNAs (miR-21 and miR-214) were induced in the anti-Thy1.1 rat model, upon transformation with growth factor-β (TGF-β) *in vitro* [149]. Blocking TGF-β signalling downstream in rat epithelial cells decreased the expression of miR-21 and miR-214 and prevented TGF-β-induced EMT by increasing E-cadherin expression and decreasing alpha smooth muscle actin (α-SMA) and collagen type I expression. Therefore, it is likely that miR-21 and miR-214 expression induced by TGF-β may contribute to extracellular matrix production and mesangial proliferative glomerulonephritis. In addition miR-21 has also been reported to play a prominent role in kidney transplant fibrosis. This suggests that miR-21 is not tissue specific, and can play a pivotal role depending on the organ or tissue. Changes in miR-21 regulation have been reported by various groups following both acute as well as chronic models of kidney injury [109,110]. During acute rejection and acute pyelonephritis miR-21 is up-regulated [139]. A study by Glowacki *et al.* [113] suggested that miR-21 by itself is a novel, predictive and reliable blood marker of kidney allograft fibrosis

### 7.3. miR-200

It has been reported that miR-200c represses ZEB1 and ZEB2 and also regulates induction of apoptosis through the death receptor CD95 [150]. This allows members of the miR-200 family to prevent TGF-β-mediated epithelial-mesenchymal transition (EMT) [151]. MiR-141 and miR-200c were down-regulated in RCC resulting in the up-regulation of their common target ZFHX 1B, which leads to the attenuation of CDH 1/E-cadherin transcription [152]. This implies that miRNAs vary in their expression due to different types of tumors and leads to different cellular pathways, such as apoptosis (Figure 2). MiR-200 family members are also involved in Wilm’s Tumor where the levels of three renal specific miRNAs (miR-192, miR-194 and miR-215) and the two members of miR-200 family (miR-200c and miR-141) were significantly decreased [153]. Here they were involved in the regulation of their common target activation receptor type 2B (ACVR 2B) through the TGF-β pathway (Figure 2). Urinary expression of miR-200a, miR-200b and miR-429 was down regulated in patients with IgAN. This suggests that they may play a significant role in IgAN, as the changes in the expression of these miRNAs correlate with disease severity and progression [70]. Another study reported that patients with minimal change disease or focal glomerulosclerosis had higher levels of urinary miR-200c than those with other causes of nephrotic syndrome [108]. Finally, the expression of the miR-200 family (miR-200a, miR-200b, miR-200c) and miR-141) is negatively correlated with VEGFA, and SEMA6A is the direct target gene of miR-141 [116]. 

## 8. MicroRNA in Renal Therapeutics

MiRNAs are believed to be potential candidates for renal therapy due to their specificity to their targets and control of cellular functions through their target genes. Although specific targets of many miRNAs are yet to be identified and their functional effects still to be established, their potential as therapeutic targets is overwhelming. A number of abnormal miRNA expressions have been implicated in several renal diseases and have been shown to have functional consequences for the disease process. MiRNAs have shown different expression patterns in different kidney diseases such as renal fibrosis, renal carcinoma (RCC), acute kidney injury (AKI), and diabetic nephropathy [70]. 

In general, there are two approaches to developing miRNA-based therapeutics, namely, miRNA antagomirs and miRNA mimics. The antagomirs (oligonucleotides) may also non-specifically bind to other RNAs, which could result in negative side effects. The therapeutic effect of inhibiting mir-208 was first described by Montgomery *et al.* [154], who showed that miR-inhibition by locked nucleic acid (LNA) modified anti-miR could protect rats from hypertension induced heart failure. For example, regulation of cardiomyocytes by miR-208 led to a decrease in cardiac contractility, possibly resulting from perturbations in the cardiac conduction system causing atrial fibrillation of miR-208a knockout mice [155]. Oligonucleotides have been widely used for inhibiting indigenous miRNA function in diseases [26,156,157]. Due to irreversible binding, miRNA is unable to be processed by RNA-induced silencing complex (RISC) or degraded. For example, inhibition of miR-24 has been reported to promote apoptosis of cardiomyocytes while decreasing the survival of endothelial cells [155]. Despite the potential challenges that face miRNA-mediated therapies, strategies targeting this technology are being explored to fight different kidney diseases.

Oligonucleotides that specifically bind to the active site in miRNA-21, inhibiting its function, have been produced and suggest that inhibitory oligonucleotides may have therapeutic potential. In both human and animal studies, it has been reported that anti-miR21 oligonucleotides accumulate in the kidney and effectively block miR-21 functions [109]. Blocking renal miR-21 expression reduces macrophage infiltration in diseased kidneys [156,157,158]. These results suggest that miR-21 also plays a role in promoting renal inflammation during kidney injury. MiR-21 has also been reported to block fibrosis in cardiovascular and pulmonary diseases [123]. Anti-miR-192 treatments ameliorated glomerular fibrosis in mouse models of diabetic nephropathy through a concomitant repression of collagen and fibronectin levels in the mesangial cells [97]. MiRNAs and their target genes represent interesting pharmaceutical targets as part of a general or personalized therapy in the future.

## 9. Biomarkers

Over the past decade, miRNAs have emerged as excellent biomarkers of kidney diseases and represent potential novel therapeutic targets because they are stable and tissue specific. Specific miRNAs play a significant role in the occurrence and progression of kidney disease. In addition, the tissue specificity of miRNA expression makes them ideal candidates for biomarkers for early diagnosis of malignancies and other diseases [159]. They exhibit an extreme high stability in formalin-fixed tissues, plasma and serums samples [160] and are present in most solid tissues [161]. Several studies confirm the fact that miRNAs are detectable in various body fluids such as serum, saliva, tears, urine and blood [110,162,163]. Their stability and presence in body fluids pave the way for the use of miRNAs as diagnostic and prognostic biomarkers for human disease. MiRNAs have been employed in the diagnosis of kidney diseases but can also be used in the prognosis and response to therapy in the near future. MiRNA signatures can be used as reliable biomarkers for diagnosis, prognosis and response to therapy. 

Perhaps most importantly biomarkers must be associated with the biological mechanisms within the disease. Multiple studies in the kidney have dealt with the tissue expression pattern in various renal diseases such as renal cell carcinomas, renal allograft and polycystic kidney diseases [164,165,166,167]. For example, a study conducted by Gottardo *et al.* [166] has identified miR-28, miR-185, miR-27 and let 7f2 as being differently expressed in renal cell carcinoma as compared to normal kidney tissue.

## 10. Prospective

The emergence of miRNAs as regulators of gene expression identifies them as obvious novel candidate diagnostic and prognostic indicators, and potential therapeutic targets. There are several major challenges in exploring the role of miRNAs in kidney diseases. To date, few studies have focused on miRNA in urine and blood as a potential biomarker for the detection of kidney injury and diseases. In rats, kidney tissue, blood and urine levels of miR-21, miR-155 and miR18a were evaluated [168]. These studies are promising and continued exploration into the possibility of circulating miRNAs as predictive factors for kidney diseases is important. Understanding the pathophysiological role of a specific miRNA in the kidney is difficult due to the fact that the kidney is composed of various types of cells and these cells may respond differentially to miRNAs in several renal diseases. MiRNA expression has been proposed to be a diagnostic marker for IgA nephropathy and lupus nephritis [71].

The recent discovery that miRNAs are detectable and quantifiable in the circulation adds further scope to their potential, particularly as evidence accumulates to support their use as biomarkers of renal disease Spector *et al.* [158] reported the use of miRNAs to differentiate four types of renal cell carcinomas (such as clear cell, papillary, chromophobe and oncocytoma) from one another. MiRNA studies in various renal diseases have shown not only that miRNA expression is differentially regulated but also that the expression pattern itself could be a useful tool for diseases diagnosis. Despite the rapid growth of information regarding miRNAs, the role of miRNA regulation of normal and abnormal kidney function is not fully understood.

MiRNA have the potential of being reliable biomarkers because they are tissue specific and stable in different biological fluids. Additionally, the discovery of the association between miRNAs and different diseases would provide potential targets for novel therapies in kidney diseases. MiRNA continues to generate new findings; a challenge for the future is to translate some of the experimental findings to potential therapeutic interventions. Furthermore, it should be noted that large-scale studies are required to evaluate the clinical value of miRNAs.

## 11. Conclusions

Rapid progress has been made on understanding the function of miRNAs in the pathophysiology of diseases, such as renal diseases and others over the last decade. MiRNAs play a fundamental role in gene regulation and have the capacity to modulate multiple gene pathways. Elucidating the roles played by miRNAs in all aspects of renal functioning remains challenging. Many studies have demonstrated the crucial roles that miRNAs exert during the progression of metabolic pathways. Their abnormal transcription has emerged as a vital regulator underlying a diverse range of renal pathogenesis. Small molecules such as miRNA could potentially lead to accurate diagnoses and the generation of novel therapeutic approaches to maintain and improve renal function after injury. MiRNAs could have great value for research into new therapeutic targets. Additionally, studies have shown specific roles of miRNAs, such as miR-192, miR-194 and miR-215 and the miR-200 family (miR-200c and miR-141) in Wilm’s tumor pathogenesis [169]. Dysregulated miRNA levels in biological fluids, such as plasma, serum or blood, could represent a new source of biomarkers in renal diseases.
